# Re-orientating health and nursing care: a qualitative study on indigenous conceptualisations of wellbeing

**DOI:** 10.1186/s12912-022-01063-1

**Published:** 2022-11-02

**Authors:** Karen McBride-Henry, Michael Roguski, Charissa Miller, Kim Van Wissen, Padmapriya Saravanakumar

**Affiliations:** 1 School of Nursing, Midwifery, and Health Practice, Wellington Faculty of Health, Victoria University of Wellington, Wellington, New Zealand; 2 Kaitiaki Research and Evaluation, Wellington, New Zealand; 3 School of Nursing & Midwifery, Faculty of Health, University of Technology Sydney, Ultimo, NSW Australia

**Keywords:** Culturally responsive, Indigenous, Kaupapa Māori, Nursing, Older adults, Pakeke, Positive ageing, Qualitative, Wellbeing, Nursing, Health Services

## Abstract

**Background:**

Health systems often fail to address the wellbeing needs of older Indigenous populations; this is attributed to a lack of knowledge of Indigenous health systems arising from a privileging of dominant western biomedical epistemologies. In Aotearoa/New Zealand, there is a dearth of nursing knowledge relating to Māori, which negatively impacts on the provision of holistic nursing care. This research explores insights and perspectives of older Māori adult’s (pakeke) perceptions of wellbeing so nurses can provide culturally responsive care and support the wellbeing of Indigenous New Zealanders.

**Methods:**

An Indigenous kaupapa Māori methodology underpinned and directed this research project. Audio-recorded interviews were conducted face to face in participants’ homes, marae (meeting house) and workplaces. Pakeke over the age of 55 participated in in-depth interviews. A total of 10 pakeke were interviewed and narratives were thematically analysed in accordance with meanings derived from Māori worldviews.

**Results:**

Wellbeing was attributed to the holistic interconnection and balancing of whānau (wider family), whanaungatanga (social connectedness), hinengaro (mental and emotional wellbeing), taha tinana (physical wellbeing) and wairua (spirituality).

**Conclusion:**

The findings offer unique insights into how wellbeing is constructed for pakeke; the results are unique but consistent with international accounts of older Indigenous peoples. Pakeke wellbeing can be supported by acknowledging existing cultural and spiritual beliefs and peer-support initiatives. Nursing models of care should prioritise Indigenous ways of knowing; this research offers nursing-focused recommendations to improve care.

## Background

The World Health Organization highlights the importance of having meaningful research to develop robust understandings of and monitor the health of older population groups [1]. A key aspect of this challenge is understanding what constitutes wellbeing for older people, inclusive of Indigenous populations, as this forms the basis of robust nursing care and health initiatives. The Indigenous population in Aotearoa/New Zealand (Aotearoa) are Māori, and this research aims to explore how older Māori adults (pakeke) perceive wellbeing.

Older adults are living longer, and nurses play a significant role in supporting older adults to live well by being health promotion advocates, providing health education and engaging in direct coordination of healthcare [2]. In response to the complex and contemporary health and wellbeing needs of the older population, nurses need to consider health initiatives that stem from the concept of wellbeing; however, definitions are frequently vague and often focus on a single aspect of wellbeing, for example, physical health. One definition of wellbeing, espoused by Adler and Seligman, is “what individuals intrinsically value” [3] (p.1); however, according to Ereaut and Whiting “wellbeing has a holographic quality; different meanings are being projected by different agents” [4] (p.5). This has led to confusion about what wellbeing means for older adults and how to develop holistic, or wellbeing, health plans that support people as they age.

What individuals value changes across the life-course, so research specifically focused on older adults’ wellbeing is important [5, 6]. Wellbeing is defined by the individual, although it is situated within family, culture, socio-political and environmental influences [3, 7, 8]. Indeed, according to Holmes [9], wellbeing can be experienced by an array of individuals who may otherwise be deemed incapable of experiencing successful aging due to comorbid chronic illnesses or disabilities. By way of example, Perry and colleagues [10] conducted a meta-synthesis of older adults’ wellbeing post-orthopaedic surgery. Their analysis identified four overarching concepts that emerged that impacted wellbeing; they were ‘loss of independence, limitations with activities and function, coping and pain, with people’s mental outlook playing a significant role in maintaining wellbeing. In addition, wellbeing researchers often overlook the need to adopt a strength-based approach to understanding wellbeing. However, nurses can learn much about wellbeing by considering Indigenous worldviews.

Within Indigenous populations, beliefs about the importance of wellbeing have existed for many years, with wellbeing considered a multidimensional phenomenon [6, 11, 12]. McCubbin and colleagues highlight that Indigenous wellbeing has a solid relational component that stems from “confidence and perceived competence to overcome adversity, respect, and be in harmony with nature and ancestors through cultural practices, the management of financial resources, family commitment, access to quality health care, and involvement in and contributing to one’s community” [11] (p.362). These authors suggest that western understandings do not reflect what wellbeing is for Indigenous peoples, so it is impossible to support their pursuit of wellbeing meaningfully without insights into Indigenous paradigms. Other researchers argue that frameworks need to be developed from the perspective of the elders to allow an understanding of how culture impacts and shapes aging [5, 6]. To this end, no Indigenous health promotion intervention(s) should occur without the involvement of elders from within the community [13].

Indigenous wellbeing frameworks rely heavily on strength-based approaches to underpin wellbeing [14]. Further, a meta-analysis of qualitative research revealed three distinct findings that described how ‘wellbeing’ for Indigenous populations might be supported in primary health care settings they are: ‘Maintaining Indigenous identities’, ‘Promoting independence’, and ‘Delivering culturally appropriate care’ [15]. This information provides insights into what is essential for Indigenous populations concerning care delivery at a systems level, but less about addressing wellbeing when working directly with older Indigenous adults.

Māori are the Indigenous population from Aotearoa. It is widely recognised that colonisation continues to have a significant impact on Māori[16–18]. In addition, it is also accepted that the health system has failed in its obligations to address the needs of Māori [19, 20]. However, the creation of culturally meaningful, strength-based research focused on wellbeing from an Indigenous, in this instance, Māori, perspective will produce evidence to support nurses in providing culturally responsive and meaningful care.

The foundational work that describes wellbeing from a Māori worldview is Durie’s health promotion framework, Te Whare Tapa Whā [21]. Within this model, wellbeing consists of four dimensions: Taha whānau (social/family wellbeing); taha wairua (spiritual wellbeing); taha hinengaro (mental and emotional wellbeing), and taha tinana (physical wellbeing) (see Table 1 for the glossary of terms used in this paper). If you remove one of these dimensions, wellbeing is damaged or cannot exist. Therefore, any care interventions need to consider all dimensions of wellbeing, or in other words, a holistic perspective.

**Table 1:** Glossary

Te Reo Māori	English Translation
Ha Taonga Tukuiho	Heirloom or heritage
Hinengaro	Mental and emotional wellbeing
Kaitiaki	Guardian
Kaitiakitanga	Stewardship
Kaumātua	Māori elders who are held in high esteem
Kaupapa Māori methodology	Māori approach to research and research practices
Manaakitanga	Kindness, respect and generosity for others
Marae	Courtyard where Māori gather in front of a meeting house
Mokopuna	Grandchildren
Pakeke	Older Māori adults
Tamariki	Children
Tāne	Male
Te ao Māori	The Māori world view
Te reo	Māori language
Tikanga	That which is right
Tīpuna	Ancestors
Taha tinana	Physical body
Turangawaewae	A place of belonging through whakapapa
Wairua	Spirit and spirituality
Whānau	Wider family
Whanaungatanga	Social connectedness
Whakapapa	Genealogy that includes leadership, rights, kinship and status
***Definitions source***:	[44, 45]

Only limited research exists that examines the wellbeing of older Māori adults when considering wellbeing as a holistic concept. Using the Māori Cultural Identity Scale, Apiti examined survey results analysed based on the concepts embedded in Te Whare Tapa Whā [22]. Findings revealed that Māori cultural identity is an important aspect of wellbeing for Māori and positively impacted their health; Apiti called for health care providers to embrace Indigenous understandings of wellbeing. However, within the context of Aotearoa, the authors were unable to locate nursing research that focused on the wellbeing of older Māori adults within a public health or health promotion setting. Another study by Oetzel and colleagues [23], examined how peer-mentoring would support positive transitions amongst older Māori adults. The results of this study demonstrate that the involvement of kaumātua (Māori elders held in high esteem) to support and provide peer-mentor support led to strength-based collaboration that supported enhanced health measures; ultimately services provided by kaumātua for kaumātua resulted in positive social connections and health outcomes. Research such as this provides important evidence for nurses developing health services for pakeke.

Contemporarily, the predominant biomedical model in which nurses work is the western model, which is considered to be inadequate for Indigenous peoples and is antagonistic to Indigenous notions of self [24]. Despite this, nurses need to understand and mitigate the impact of colonisation and provide meaningful care for Māori. To this end, nurses in Aotearoa have worked to support nurses to provide culturally safe care [25, 26], which informed global discussions on how nurses could better meet the needs of Indigenous populations. More recently, Māori nurses have been problematising the lack of progress towards equity for Māori because the understanding of cultural safety as defined by Ramsden, has been diluted by non-Māori [27, 28]. Wilson suggests that a lack of progress towards cultural safety has occurred because non-Māori have engaged in a transliteration process that has distorted and changed core tenets of cultural safety. As a consequence, commonly accepted understandings do not reflect Ramsden’s original intent [27, 28]. Instead, “nursing has languished in unfulfilled rhetoric and is complicit in the long-standing Māori health inequities, upholding systemic practices deemed to be racist…” [27](pg.30).

However, for nurses seeking a different approach to care provision there is little research to inform and shape the nursing care for older adults that embraces a holistic perspective of well-being. To construct knowledge, nurses often need to piece together small portions of knowledge from wider studies, including cohorts of non-Māori respondents. For example, Wiles and colleagues [29] conducted a study that examined caregivers’ experiences of older adults who are dying. Their sample included Māori and non-Māori participants, and although this study helps provide insights, it is not explicitly focused on the needs of Māori, as such, the transferability of the findings are limited.

There are many well regarded definitions of nursing. One example was created by the International Council of Nurses, which states that “Nursing includes the promotion of health, prevention of illness.”[30] Another example is offered by Diers; “Nursing is two things: the care of the sick (or potentially sick) and the tending of the entire environment within which care happens” [31] (p.1). These definitions highlight the holistic nature of nursing care; this is of significance as conventional Western nursing has failed to recognise the holistic nature of health instead being subservient to a myopic biomedical fragmented approach to care provision.

In addition, according to Whitehead [32], nurses fundamentally seek to deliver care that is holistic and encompasses health education and promotion. Indeed, nursing practice draws heavily on a strength-based lens when planning care interventions, including wellbeing promotion. Undoubtedly, care planning is vitally important across the lifespan, and nurses “seek to empower individuals, families, groups and communities…” through their health promotion activities [32] (p.39). Therefore, wellbeing promotion fits comfortably within the remit of nursing practice. However, to promote wellbeing as a nursing task, we must understand how those we care for conceptualise wellbeing; a call issued by other researchers [33].

In an ideal world, Māori nurses would care for Māori; however, the number of Māori nurses does not proportionally represent the number of Māori within the population of Aotearoa. In Aotearoa, 7.6% of NZ nurses are Māori, 58.5% are NZ European, with expatriate nurses constituting the remainder of the workforce [34]. Indeed, most of the nursing workforce is non-Māori (92%) [35]. Due to the disproportionate number of non-Māori nurses, nurses need to respond appropriately and provide culturally-responsive and meaningful health care.

It is worth noting that migrant nurses must undergo cultural training before registering as nurses in Aotearoa; however, these nurses are potentially disadvantaged because they have not worked with Māori or other Indigenous populations before. Also, recently migrated internationally qualified nurses are most likely to gain initial employment with older adult service providers such as residential care facilities [36], potentially confounding the lack of culturally responsive care provided to Māori within older adult health care settings. Nurses need research evidence, skills, and resources to care for older Māori adults; this is especially true given the health system’s overall failure to deliver equal health outcomes. Nurses need research that can re-shape the provision of care for Māori. With this overarching aspiration, this research was conducted, so nurses can better understand what constitutes wellbeing for pakeke, or older Māori adults, in Aotearoa.

## Methods

A kaupapa Māori research methodology underpinned this research. This methodology is derived from distinctive cultural epistemological and metaphysical foundations that frame and structure how Māori think and practice [37, 38]. The methodology is inherently political and arose in response to the continued marginalisation and silencing of Indigenous voices and knowledge. At its foundation, kaupapa Māori research methodology actively positions te ao Māori (Māori worldview) as central to the gathering, analysis and reporting of narratives. As such, Māori values and approaches are embedded and drive the research process [16, 17]. Naturally aligned with this premise, the reclamation of Māori epistemology stresses that research with Maori is undertaken by, with and for Māori using approaches based on te ao Māori, that draw upon mātauranga Māori (Māori knowledge), Māori values and tikanga [39–42]. Ethics approval for this study was granted by the Victoria University of Wellington Human Ethics Committee (approval number 26,394). A kaupapa Māori purposive sampling recruitment technique was used and was entirely driven by the Māori research team member (started 02/2019). All participants gave verbal and written consent before the semi-structured interviews. Ten face to face interviews, of 45–70 min duration, were conducted by the Māori researcher, with a total of five pakeke tāne (older adult men) and five pakeke wāhine (older adult women) involved. The participants were from both urban and rural parts of Aotearoa.

Kaupapa Māori methodology embraces qualitative methods that support the purposeful telling of stories to understand human experience and describe the meaning that these hold for people [16]. Narratives were analysed in accordance with a qualitative thematic analysis embedded within a kaupapa Māori research methodology [39–41, 43]. The approach involved an initial comparative analysis, which led to the development of themes and subthemes; an approach in keeping with the method described by Wilson and colleagues [42].

The interviews were conducted in English and te reo Māori and metaphors used throughout (see Table 1 for a glossary of terms). MR is a native te reo Māori speaker and supported the translation in agreement with the kaumatua involved in this project. The interviews were audio-recorded and subsequently transcribed verbatim and returned to the participants. The interview transcripts were analysed following te ao Māori embedded principles as identified by the participants. The pakeke participants involved supported the development of themes, and the findings are presented in this article reflect their wisdom and narratives. The final version of the analysis was critically assessed, within te ao Māori, by one of the participants, who holds kaumatua status and is regarded as an expert in Indigenous conceptualisations of self and wellbeing. The (non-Māori) research team members provided guidance and theoretical support to frame the findings within an international and national nursing context, and they were not involved with the recruitment or analysis.

## Results

The experiences and worldviews gathered during this research were analysed into themes that reflect coalesced narratives that focused on the balance between the various theme that contributed to wellbeing. These themes were: wellbeing, whānau (wider family), whanaungatanga (social connectedness), taha hinengaro (mental and emotional wellbeing), taha tinana (physical wellbeing) and wairua (spirituality). The findings are presented as separate themes, but the concepts within the themes are indivisible and interlinked. Instead, the concepts described in the findings examine the various facets that encapsulate and provide the basis for wellbeing for Māori pakeke.

### Wellbeing

The use of ‘balance’ was common across participants’ wellbeing narratives. Balance was used to refer to the simultaneous and holistic presence of elements that underpin the individual’s engagement with life, no matter what challenges the individual might be facing.

I find that if I’m not training physically, my mind doesn’t work properly. There’s an imbalance there … I find that if in my own personal space, if I’ve got a balance around physical and mental activity, I can pretty much deal with most things or anything, really. It’s not gonna trip me up… in general, I find that if my whānau are fine, if there’s a problem in the whānau, if I’m still working on the physical and the mental wellbeing consistently, then I find it’s easier to deal with. (Tāne)

Importantly, these elements were embedded within an overarching discourse of the individual’s positioning and responsibilities across time; this can be appreciated in that the self was commonly referred to in deference to one’s tīpuna (ancestors) whilst simultaneously viewing the individual’s responsibility to prepare a pathway for following generations. These time-related positionings acknowledged Ha Taonga Tukuiho, the individual, as a treasure that has been passed down. As such, wellbeing discussions were commonly framed in consideration of past generations and a sense of responsibility for the next. Being mindful of past and future generations, framed the individual as kaitiaki (guardians), with an obligation to honour those who have gone before while preparing future generations; a positioning that provided a deep sense of purpose.

I reflect upon tīpuna, who really struggled for this life, and it helps me to motivate my own wellbeing… I think that there’s a purpose. That there’s a purpose that we’re gifted this [life]. And it’s how you look after the vessel, so that you can do the longevity. [It is} kaitiakitanga [stewardship]. It’s innate, really. Because that’s the worldview that I’ve been brought up in. (Wāhine)

Wellbeing was attributed to the holistic interconnection of whānau, whanaungatanga, hinengaro, taha tinana and wairua. Each element was interwoven throughout participant narratives.

### Whānau (wider family)

Participants placed considerable emphasis on whānau as an integral component of wellbeing. On one level, the importance of whānau was raised as the basis of individual identity. As such, participants expended considerable energy assisting whānau attain the highest degree of fulfilment, the attainment of which provided a foundation to the individual’s wellbeing. The importance of whānau wellbeing was discussed in relation to balance whereby whānau wellbeing acted as a foundation to other aspects of the individual’s life. In this sense, challenges within the whānau were discussed as negatively impacting the individual’s wellbeing.

I think, most importantly, is that there’s an ambience of calm and tranquillity within the whānau. I think if you see your children, being adult or whatever, achieving, there’s less stress on you … The most important part is that if your own backyard is tranquil and everything is running smoothly, that transfers itself into the other domains that you go into, like work or whatever. That’s what I see as being the centre of things. (Tāne)

Linked to the centrality of whānau, considerable joy was described from being able to observe whānau thrive and watching the younger generations grow.

I think the other thing is that if you’ve experienced love. It’s something that continues to assist you to thrive even further. I’m talking about the love of meeting someone who’s your soulmate and the love of watching your next-generation growing … I’m talking about your moko [grandchildren] and seeing those same things that you felt at your own parents, and your grandparents suddenly start manifesting themselves in your mokopuna [grandchildren]. I love that. (Tāne)

I find my mokopuna (grandchildren) as being my motivators… the little people that keep your childlike voice alive. (Wāhine)

Roles associated with older adulthood facilitated the position of nurturer and observer. For instance, one participant described how his role had changed from that of a more authoritarian leader, as one attempting to enforce whānau compliance, to one of a connector. Embedded within this role change is one of ‘nurturer’, whereby the individual is better positioned to enjoy watching whānau develop.

I must admit, I was a bit of a bully in my family when I was younger, but now I’m the connector across my siblings and cousins … That’s another place where I get my source of inspiration from. (Tāne)

In addition to mokopuna, adult whānau members were referred to as a primary element of wellbeing, providing a sense of connectedness and unconditional love.

Usually, about once every two/three months, we’ll all meet at me brother’s. We’ll stay up all night and we’ll just talk about silly things. I love that. We’re all together, laughing about the stupid things we did as kids … It’s like I’m connected to someone. I love someone and they love me unconditionally … That’s powerful. It is. We’ve always been close. We did some funny things. (Tāne)

Okay. I definitely need my family. Yeah, I can’t imagine what I would be like if I didn’t have a family to connect to, or if I didn’t have a connection to my family on that -- I just couldn’t imagine what that would be. (Wāhine)

The importance of whānau, and especially older whānau members, was noted as providing an affirmation of tikanga (that which is right); affirming manaakitanga (the process of showing respect, generosity and care for others) and the application of kaitiakitanga (stewardship), whilst sustaining whakapapa (future generations).

They’re reminders of tīpuna for me. Looking at them, listening to them. They affirm a lot of my thinking around how we look after each other, how we apply tikanga in our processes, how we apply the tikanga of the manaakitanga, how we apply kaitiakitanga; how we retain and sustain whakapapa. So, they’re the affirmation for me in regards to the people that I find life-giving for me. Whakapapa - and my family, etc., are really good, there’s also good friends and colleagues, and particular ones that you rely on the most. Where you’ve got lots of commonalities. It’s also good to have those you don’t have things in common with, Essentially those would be what those people look like. (Wāhine)

Whānau discussions were also underscored as a vital investment in the future. Rather than focusing on material wealth, emphasis was placed on investing in whānau. Such investment-related discourse is founded on a principle of reciprocity, where the younger generation assists older members.

I have this crazy view that I’m going to put all my investment in maintaining good healthy relationships with my whānau instead of putting it in the bank. When we die, the investment is, if I’ve looked after the healthy relationships in our whānau, they’ll be looking after me. Very much like my partner and I looked after her father. Just like we looked after my parents… You should be investing in your loved ones around you. (Tāne)

### Whanaungatanga (social connectedness)

Social connectedness was discussed on three levels. On a basic level, social connectedness was described as necessary because of a belief that a lack of connectedness can be detrimental to the individual’s wellbeing. In this sense, social connectedness was furthered as a means of extending one’s self – exposing the individual to new people, ideas and experiences. Connectedness was also framed within a combative discourse, acknowledging that isolation can have detrimental effects.

Don’t live an isolated life. Socialise. Socialising brings other things into your life. It brings information, it brings new friendships. You meet people that you wouldn’t normally associate with. All of those things, combined, I see as being imperative. (Tāne)

Next, social connectedness was discussed as a means of support: the existence of a social network providing support in time of need or crisis.

You’ve got all the support there. All the supports there can help you through whatever you’re going through. It’s like mental health. Don’t be scared to ask for help. (Tāne)

I revolve around a family, there’s a negative thing about being involved in a whānau is that you have a lot of tangi [funerals]. There’s a lot more issues to deal with. But the plus side of it is there’s more of an instant support network there too. There’s people that you value and they value you. It’s a good stocktake. I have some mates that I keep in touch with, even today, but in my family, I also have seen things that have emerged between the generations. The younger generations, who are now parents themselves, doing very similar things to what me and my brothers were doing, are now starting to enter that fray of moving out of just looking after your own family network. (Tāne)

At a deeper level, wider social connectedness provided participants with an opportunity to express manaakitanga, or the process of showing generosity and care for others. Similar to a sense of joy and fulfilment, participants described watching their whānau thrive, and this communicated a similar sense of satisfaction from helping others. Manaakitanga was referenced both tamariki (children) and adults. Importantly, the sense of fulfilment cannot be separated from other wellbeing elements of wairua, taha hinengaro and taha tinana.

The whanaungatanga that’s here [marae]. Interacting with children, helping them with gardens or helping them do projects. You can always be busy here, and that’s why I love it, cos the mental part helps the physical … I’m in the moment with them, and all this is there. I’m in the moment with them and I’m enjoying it mentally. The physical [physical impairment] parts are nothing. That physical thing’s [physical impairment] gone because you’re on a wairua level with them. You’re all one. That’s powerful. I love it. The look on their faces, and adults helping them. All that comes into it. There’s nothing like it. (Tāne)

Helping others get to that place of like they’re thriving… my day job is working with people affected by addiction, whether they’re the whānau or they’re the consumer, so it’s working alongside people that are trying to cope with drugs in their lives, form new relationships or help them repair broken relationship with whānau. It’s always a positive - it’s always good for me when you see them getting somewhere… I think something about this sort of work, it’s kinda like soul food. It makes you feel good when you finally see one of your clients leave and they’re leaving in a much better place. (Wāhine)

### Taha Hinengaro (Mental and emotional wellbeing)

The importance of having a sense of purpose was common across narratives and was framed as contributing to an individual’s life engagement and overall happiness. Purpose was inextricably linked to having a sense of being valued through the individual’s ability to contribute in a meaningful way.

For me, it’s having a sense of doing something that’s worthwhile… And I think I thrive off that … So that sort of sense of being of value, I suppose. (Wāhine)

Hinengaro was also reflected in the importance of maintaining a sense of ‘peace and harmony’. While harmony was discussed as being reliant on other wellbeing elements being in balance, considerable emphasis was placed on the importance of the one’s attitude and ways of perceiving life events to avoid internalising stressors.

With increasing life experience, participants described having learnt how to cope with significant life events and stressors such as the death of loved ones. Instead, the importance of maintaining peace and harmony concentrated on more minor stressors that have a cumulative eroding impact.

For me, the major things were real huge. You can handle them, cos everyone has them. You get a disaster and that, they’ll knuckle down and they brace themselves. What fucks people is everyday life. It’s just that little bit of chippin’ away, chippin’ away, chippin’ away and then one day they’ll lose the plot and everyone’s going, whoa, where did that come from? (Tāne)

Across participants, everyday stressors generally took the form of negative encounters with people. As such, overt negative-avoidance strategies were employed, such as removing oneself from situations or people deemed as negative. Simultaneously, clear boundaries were communicated about interpersonal relationships and the importance of not being around those considered unfavourable.

No negativity. If there’s negativity, I’ll just walk out … Because I don’t like negativity. I can see it, but if I can avoid it, I will… (Tāne).

Peace and harmony [are important to me]. I don’t like arguments. Cos I’ve seen a lot of people destroyed by anger and alcohol-related stuff and all that. Drugs and all that. That’s just not me. (Tāne)

Seniority in the workplace or the independence that stems from retirement facilitated the individual’s freedom to disengage from negative situations and people. Others described a shift in perspective towards conflict as an outcome of maturation. Such shifts resulted in reduced anxiety, anger and stress. Cape Reinga was referenced as a metaphor for conflict, drawing on the imagery of the turmoil arising from the clash of the Tasman Sea and the Pacific Ocean. With maturation, the participant described shifting from being embroiled in the turmoil to adopting a lighthouse perspective.

But where I got my peace from there – where it felt like a thriving space – just being there at papaki tu ana nga tai ki te Reinga - the crashing of the waves. When you go out to Cape Reinga and you see where the two oceans meet. I was right down the midst of where the waves were crashing. Then, suddenly, it seemed like overnight, it flipped. That’s probably one of the reasons why I left [the university]. I suddenly found myself up where the lighthouse was and looked at the wider expanses of what was occurring. (Tāne)

Notably, life events, such as sudden acute illnesses, played a substantive role in facilitating a reprioritisation process for many participants, resulting in a cognitive shift and a need to place the self and whānau as the priority over career and other activities that led to what one participant referred to as ‘contestations’. Such shifts are significant in that participants generally welcomed supportive roles but tended to be wary of positions that could result in stress arising from conflict.

I’m amazed now at how I can just sit back and not have to take the front seat. But at the same time, I’ve got [a] valuable voice … my vision’s shifted now. When you’re in the fray and building a family and building your career and your credibility in the workspace and building your expertise in sport … But then, suddenly, when you have an event or events or life changes, these matter very little to me. (Tāne)

In addition to attitudinal shifts in how to perceive stressors, participants described maintaining peace and harmony through ‘alone time’. The need for alone time can be appreciated in that all participants reported being heavily involved in either paid or unpaid work as well as a host of whānau and community commitments and responsibilities.

Sometimes you need to think about yourself and you need to give yourself a little love, at the end of the day you become an empty shell. Cos you’re giving it out to everybody, but then you leave yourself out. So, for me, learn to love yourself. It doesn’t mean you gotta be arrogant about it. It’s to be humble. (Wāhine)

‘Alone time’ was strongly linked to wairua (spirituality) and was reflected in reliance on nature, returning to one’s Turangawaewae (a place of belonging through whakapapa). The physical world was described as especially important in combating sadness, stress or simply as a form of communion.

If you get really caught up or you’re feeling like you’re a little bit stressed or something. I find a weekend at home [Turangawaewae] just brings me straight back to where I should be. The sea’s a big part of it for us, we come from the sea. There’s something about a tranquillity that comes from just sitting on the beach and breathing in the air and listening to the waves and just watching the beauty that is the ocean. (Tāne)

In other situations, peaceful engagement was achieved through lone activities such as reading, puzzles, riding one’s motorcycle and, in one case, playing the pokies.

If you keep giving out love, like you give out love to your brothers, sisters, cousins and friends- at the end of the day, do you ever think about yourself? You’re in the same picture. You’re not out of it. You need to think about you, which I do quite a bit. (Wāhine)

I always take time for myself, not much on most days, and I do Wordscape … I read a lot … I ride my bike… I might ride with others but I’m on my own and I’m thinking … Because I’m always around people. So, finding that time just to drift into the world - where I just get my mind to just relax. I enjoy it. (Wāhine)

Markedly, the maintenance of peace and harmony was facilitated by a sense of increased resilience, learnt from overcoming adversity throughout participants’ lives. In this vein, attitudes towards adversity were discussed as a mechanism of resilience. Notably, attitude was discussed as a protective factor to maintaining one’s wellbeing and was linked to the co-occurrence of whānau and wider support.

From my family upbringing, it was like, “You’ve got a choice here, you either wallow in it, or it is what it is, and you need to get on.“ Things did happen, and yes they were sad and tragic, but you had some choices as to how you dealt with that [with the death of my partner]. So, I suppose I drew on that strength, that okay this has happened, I’ve got some choices here, I could fall apart or I could just get on. (Wāhine)

Finally, mental wellbeing was discussed in terms of the importance of being mentally active. Axioms such as ‘use it or lose it’ were enlisted to reflect a combative discourse of resisting anticipated decline.

I think mental wise, to me if you’ve got your brains active and that, less chance of atrophying. (Tāne)

### Taha Tinana (physical wellbeing)

Physical wellbeing was commonly linked to nutrition, physical exercise and the need for regular health checks. While some participants stated that physical activity had featured prominently throughout their lives, the need for physical exercise and improved diets had arisen as a result of an acute health episode or the diagnosis of a chronic condition. Similarly, the need for regular health assessments had been informed by the individual’s own health episodes or as an outcome of vicarious learning. Importantly, physical activity was described as a central element of individual wellbeing as activity alleviated many of the presenting symptoms, as insufficient activity resulted in impeded mobility.

Some days I’m in chronic pain, but I try and walk it off. Keep moving. I can move my arm and all that, but it’s that pain. Even pills, I’m not a very pill popper. I get told off by my doctor all the time, cos I don’t take my pills, blood thinners and all that. (Tāne)

On a deeper level, participants discussed engaging in physical activity as inactivity was viewed as a precursor to an early demise, a position similar to a combative discourse of fighting anticipated mental decline.

I think it’s the fear of, if I don’t do nothing, that’s it - check out time. You’ve gotta keep going. (Tāne)

### Wairua (spirituality)

Wairua was often used to describe spiritual connections and meanings arising from connections to or simply being one with the environment. As such, wairua is inextricable from the various wellbeing elements of whānau, whanaungatanga, tinana and hinengaro. While interwoven throughout the elements, wairua was discussed as an essential element of wellbeing in itself. Wairua was a sense of grounding the individual holds in having a purpose.

My faith has been another very strong space. The faith, and also the cultural values. Those have really worked in harmony for me. Because I value the fact that we’re here for a reason. It’s just not something that’s been put together by the drop of two atoms. That’s how I see the world. I’ve had too many of my loved ones pass away to even contemplate the fact that I’ll never see them again. (Tāne)

Groundedness or having a sense of foundation was commonly referenced.

I need to have a sense of feeling my feet underneath me. That means, you know, if I’m able to walk I can jog. If I can jog, I can climb. If I can climb, I can do my mahi [work]. The sense of knowing that I’ve got my feet under me, and I’ve got my head screwed on. (Tāne)

On a daily basis, wairua connections manifested through prayer and reflection, setting daily intentions, reflecting on the individual’s position and relying on wairua for protection. Importantly, it is not possible to compartmentalise wairua. In some instances, wairua reflected western conceptualisations of prayer, whereas, in other situations, wairua was discussed in relation to one’s tīpuna (ancestors) or simply being one with nature.

It may sound silly, but before I go to sleep, I say, ‘Thank you house, but don’t let anyone in here that shouldn’t be here.‘ Cos the house is covering me. Those are the things I think about, is where I’m at and making sure that I’m alright, and the house don’t get burnt down while I sleep. I think it’s the spiritual aspects of us as a Māori … I look at those things. (Wāhine)

I’m an avid reflector. Processes like starting a day, finishing a day, being close to nature in other ways, not just for physical exercise, but like watching things grow. Being in the garden. I’ve also done those things. I’ve enjoyed looking after my home. I think the second part inside that is that I also get solace in my soul. Not just through religious reverence and things like that, but also the quiet moments. (Tāne)

## Discussion

The concept of wellbeing is frequently presented as several fractured and distinct concepts. For example, physical wellbeing as distinct from mental wellbeing[46, 47]; this research highlights that pakeke, or older Māori adults, consider wellbeing in a balanced and holistic manner, which is consistent with the perspective of other Indigenous cultures that describe wellbeing as an interdependent and multi-faceted concept [6, 11, 12, 16, 48]. This exploration has revealed a paradigmatic lens of pakeke, offering unique and undocumented insights into their lifeworld. It is essential that nursing care initiatives, whatever the setting, reflect the population’s needs; without knowledge and fusing of horizons [49], it is impossible to understand the lifeworld of the people we work alongside within a nursing context. This research begins to untangle what matters to pakeke when conceptualising wellbeing, which incorporates a balance between the physical, spiritual, mental wellbeing and family. Notably, the findings of a holistic understanding of wellbeing will be of significance to all older populations.

To maintain balanced wellbeing, the pakeke who participated in this research indicated that contributions to whānau and Iwi are important. Pakeke described how when they contribute to whānau and Iwi, their physical pain and disease are minimised; this stands in contrast to how others have described the impact of pain as a phenomenon that separates and creates barriers between the sufferer and others [50]. For pakeke, these intentional spaces of connection and contribution through whānau and Iwi means that strength and resilience are built and enable pakeke to engage meaningfully within their lifeworld. Such insights are crucial for nurses supporting pakeke who, for example, live with chronic pain, as care initiatives that lead to a contribution to whānau and Iwi will support wellbeing and access to a lifeworld of connection. Taking these aspects of wellbeing into account, when developing nursing care activities, will ensure there relevance to the individual and whānau . For example, whānau should be included in wellbeing activities and building connections to ancestors and future generations should also guide nurses planning.

For the pakeke who participated in this research, connectedness with whānau and whakapapa provides a deep level of purpose; this stems from being deeply rooted with both the land, ancestors and younger generations, and are also discussed by others [16, 17, 22, 56]. This knowledge supports the idea of ‘whānau-centred’ care versus traditional western person-centric approaches. Supporting ways to keep whānau connected through nursing initiatives is imperative, without which, equity in health service delivery is unachievable. This idea is also supported by other researchers working with Indigenous people groups [48]. In addition, nursing interventions that support connectedness at a community level are likely to be more effective than interventions focused on the wellbeing of an individual pakeke [48, 56]; this means that community should be at the heart of any wellbeing initiatives to support wellbeing. In a health-specific setting, focusing on ways to involve the whānau is vital for the wellbeing of pakeke; one strategy would be to involve whānau in all nursing care encounters.

Adopting a larger and less tumultuous view of life by taking some time to reflect was helpful to the participants in providing perspective on life in relation to one’s life circumstances. One participant said he took himself *“up where the lighthouse was and looked at the wider expanses of what was occurring”;* a metaphor re-counting how he separated himself from the immediate issues and gained perspective of the greater whole, i.e. whānau-centred issues. For this participant group, reflecting on their life purpose through their whakapapa and whānau created a strong sense of meaning that positively supported their wellbeing. From a nursing perspective, we can encourage meaningful reflexivity to promote wellbeing by providing opportunities to help further the individual’s cultural and spiritual beliefs and through peer initiatives, such as those described by Oetzel and colleagues [23]. This approach has been shown to have a mitigating and protective effect against depressive symptoms amongst older adults [51].

In addition, participants in this study recognised the importance of keeping their minds and bodies active. They sought ways to ensure that these aspects of their wellbeing were cared for; the phrase ‘use it or lose it’ used by one participant captures this sentiment. The ways in which the participants tended to these aspects were by focusing on the physical environment, for example, marae and their wider whānau. Through helping others, pakeke strengthened their wellbeing; coupled with this, participants’ identified the need to take time and care for one’s spiritual self, which helped maintain taha hinengaro. These findings offer unique and previously undocumented insights into the lifeworlds of pakeke. And this is especially formative in light of previous research on positive pressures expereinced by older Māori in regard to wider whānau committments [11, 12, 52].

The participants in this research talked openly about the processes they engage in to ensure wellbeing and foster resilience. Resilience is demonstrated by being open to learning from life challenges, maintaining awareness and connecting to wairua, and also learning from the struggles of others. This approach to developing resilience stems directly from their unique worldview. Resilience has been described as the process of reorientation following significant disruption; for Māori, this process of developing resilience would include the multigenerational impact of colonisation [53]. This research enables an understanding of lived experiences and informs culturally responsive nursing initiatives. Future research should embrace whānau-centric approaches to break down silos/misunderstandings and re-orient care. As nurses, we need to change the narratives around people that we work alongside so that stereotypical portrayals do not define people; this work contributes to the re-framing of what it is to be an older Māori adult.

Approaching health in a reductionist manner, in which physical health is conceptualised as separate from the other dimensions of health, does not help address the wellbeing needs for pakeke. The findings from this research have emphasised the benefits of understanding wellbeing through the Māori worldview, where body parts and aspects of health are not conceptualised as individual entities. The findings also inform nursing care planning and management. Considering the sum of physical, social, spiritual, cultural and environmental needs is critical to providing culturally meaningful nursing care; this type of nursing care holds to the previously mentioned[31] definitions of nursing[30, 54].

Nurses have a critical role in ensuring that the health system is responsive to Māori. This aim could be supported by advocating for the inclusion of Māori in the development of nursing services and models of care. In addition, whānau need to be involved in care planning and provision, and there is a need to spend time developing relationships with whānau. This research gives insight into the priorities held by pakeke of their health and their whānau. Nurses can create spaces where different ways of knowing are valued and incorporated to enable intentional and responsive care, which is a global issue (see also[33]). Nurses need to acknowledge and accept that pakeke are experts on their wellbeing and maintain their health and resilience. A lack of knowledge on the part of non-Māori of a Māori worldview, means that this research can offer the reader vicarious insights. It can help support nurses to deliver culturally responsive care in meaningful ways. Laccos-Barret and colleagues have provided an excellent example of what a non-racist strength-based nursing care entails, beginning with a thoughtful processing of accepted bias and racist beliefs patterns [55]. A strength-based approach with a focus on Indigenous people’s strengths and potential [56]; this research further contributes to the call for the provision of inclusive and strength-based nursing care in Aotearoa, based on a Māori context and agenda.

### Limitations

As with all qualitative research, there are limitations to this study. The number of participants is small and cannot be extrapolated to represent a whole population group. However, that is not the aim of qualitative research. Instead, researchers that employ these types of methodologies seek insights into participants’ life experiences. Through surveys, future research could test the applicability of these findings with larger population groups.

## Conclusion

This research sought to understand the wellbeing of older Māori (pakeke). Whakapapa connections provided participants with a deep sense of purpose. The participants engaged in numerous practices to maintain and strengthen their wellbeing actively seeking opportunities to keep their minds and bodies active, understanding that abilities can be lost if not routinely nourished. The findings from this research offer unique and novel insights into how older Māori adults stay well. Nurses seeking to provide culturally responsive care can learn a great deal from the narratives gifted as a part of this research. Nursing models of care and care planning should prioritise Indigenous, and in Aotearoa, Māori, ways of knowing; this research offers the opportunity to begin this journey.

## Data Availability

The de-identified datasets used and/or analysed during the current study available from the corresponding author on reasonable request.
